# The Ionic and Hydrophobic Interactions Are Required for the Auto Activation of Cysteine Proteases of *Plasmodium falciparum*

**DOI:** 10.1371/journal.pone.0047227

**Published:** 2012-10-16

**Authors:** Srinivasan Sundararaj, Deepak Singh, Ajay K. Saxena, Kapil Vashisht, Puran S. Sijwali, Rajnikant Dixit, Kailash C. Pandey

**Affiliations:** 1 Host–Parasite Interaction Biology Group, National Institute of Malaria Research, Indian Council of Medical Research, Dwarka, New Delhi, India; 2 Structural Biology Laboratory, School of Life Sciences, Jawaharlal Nehru University, New Delhi, India; 3 Centre for Cellular and Molecular Biology, Hyderabad, India; Stanford University, United States of America

## Abstract

The *Plasmodium falciparum* cysteine proteases falcipain-2 and falcipain-3 are major hemoglobinases and potential antimalarial drug targets. Our previous studies demonstrated that these enzymes are equipped with specific domains for specific functions. Structural and functional analysis of falcipains showed that they have unique domains including a refolding domain and a hemoglobin binding domain. As with many proteases, falcipain-2 and falcipain-3 are synthesized as inactive zymogens. However, it is not known how these enzymes get activated for hemoglobin hydrolysis. In this study, we are presenting the first evidence that salt bridges and hydrophobic interactions are required for the auto activation of cysteine proteases of *P.falciparum*. To investigate the mechanism of activation of these enzymes, we expressed the wild type protein as well as different mutants in *E.coli*. Refolding was assessed by circular dichroism. Both CD and trans activation data showed that the wild type enzymes and mutants are rich in secondary structures with similar folds. Our study revealed that prodomain-mature domain of falcipain-2 and falcipain-3 interacts via salt bridges and hydrophobic interactions. We mutated specific residues of falcipain-2 and falcipain-3, and evaluated their ability to undergo auto processing. Mutagenesis result showed that two salt bridges (Arg ^185^ - Glu ^221^, Glu ^210^ - Lys ^403^) in falcipain-2, and one salt bridge (Arg ^202^-Glu ^238^) in falcipain-3, play crucial roles in the activation of these enzymes. Further study revealed that hydrophobic interactions present both in falcipain-2 (Phe^214,^ Trp^449^ Trp ^453^) and falcipain-3 (Phe ^231^ Trp ^457^ Trp ^461^) also play important roles in the activation of these enzymes. Our results revealed the interactions involved in auto processing of two major hemoglobinases of malaria parasite.

## Introduction

Malaria is a very important parasitic disease, and *P.falciparum* is the most virulent human malaria parasite causing 880,000 deaths per year worldwide [1]. A number of drugs are currently available to treat malaria [2] but treatment is getting complicated due to drug resistance of parasites. Recently, drug resistance against the new effective drug, Artemisinin has been shown [3], [4], and new effective drugs are needed to treat this fierce disease. Therefore, the development of other classes of effective anti-malarials, especially compounds that act against novel biochemical targets, is required. To develop such compounds, it is very important to characterize the structural and biochemical features of new drug targets.

Potential new targets for the development of novel antimalarial drugs are the papain-like cysteine proteases. Previous studies have shown that inhibitors of these proteases blocked parasite development *in vitro* and cured mice infected with the malaria parasites [5], [6]. *P. falciparum* has four such proteases that are known as falcipains; falcipain-2 (FP2) and falcipain-3 (FP3) appear to be the principle food vacuolar hemoglobinases [7], [8], [9], [10], [11], [12]. Individual disruption of FP1 and FP2 genes did not affect the erythrocytic stage parasite development. However, FP2 gene disruption led to the accumulation of undegraded hemoglobin in the food vacuole, and increased susceptibility to cysteine and aspartic protease inhibitors, indicating that FP2 is a major hemoglobin degrading protease [10]. On the other hand, disruption of FP3 could not be achieved, but the gene could be replaced with a tagged functional copy, suggesting that this enzyme is essential for erythrocytic parasites [11]. Thus, among falcipains, FP2 and FP3 are the major proteases and may be promising targets for chemotherapeutic drug development. Hence, biochemical characterization of FP2 and FP3, including elucidation of important functional properties that are different from those of host proteases, is essential.

In contrast to other papain family proteases, falcipains have unique functional domains such as refolding and hemoglobin-binding domains [12], [13], [14]. The N-terminus of the prodomain is responsible for targeting to the food vacuole [15] and the C-terminus of the prodomain is required for inhibition of falcipains [16]. Short N-terminus extensions of the mature domains of falcipains mediate folding into active forms and a C-terminal insert in FP2 mediates its interaction with hemoglobin and subsequent hydrolysis [12], [13], [17].

It is not known how falcipains undergo processing upon reaching to the food vacuole. Most of the processing studies have been conducted on the human cysteine proteases cathepsins, but the basic mechanism is not fully understood. During synthesis of preproenzyme of cathepsin, the prepeptide is removed during entry to the ER, and the procathepsin is transported to the lysosome, where an acidic milieu triggers cleavage of the prodomain by the cognate mature domain releasing free mature protease for action [18], [19], [20]. Although proteolytic removal of the propeptide is autocatalyzed by the cognate mature domain in most of the enzymes, it can also be accomplished by the action of other proteases.

Falcipains are also produced as inactive zymogens and likely undergo processing in the acidic food vacuole [8], [9], [21], where active enzymes hydrolyze hemoglobin. Studies with protease inhibitors have suggested that FP2 and FP3 are processed by auto hydrolysis [8], [9], [21], but the mechanism has not been fully explored. Our study elucidates the mechanism of activation in these enigmatic proteases. We report here that salt bridges and hydrophobic interactions are crucial for auto activation of malarial cysteine proteases, falcipains.

## Experimental Procedures

Restriction endonucleases and polymerases were from Fermentas, and ligases from Invitrogen, oligonucleotides were synthesized at Eurofins. All DNA fragments were amplified from the pTOP-pro-FP2 plasmid, and pTOP-pro-FP3, which encodes the complete FP2 and FP3 genes, respectively, as described earlier [7], [8].

### Cloning, Expression, and Refolding of different Constructs of Pro-FP2 and Pro-FP3

All DNA fragments were amplified from the pTOP-Pro-FP2 plasmid, and pTOP-Pro-FP3, which encodes the complete FP2 and FP3 genes, respectively, as described earlier [7], [8]. The constructs coding for both wild type enzymes were PCR amplified using the primers listed in table 1. The DNA fragments were digested with *BamHI* and *HindIII* for pro-FP2 construct, and *BamHI* and *SalI* for pro-FP3 construct. The fragments were gel purified and ligated into the expression vector pQE-30 (Qiagen). The constructs were transformed into M15 (pREP4) *E.coli* expression cells.

**Table 1:** Sequences of the primers used in this study, mutated sites are in bold type.

PRIMER	MUTANT	SEQUENCE(5′-3′)
**FP2 E-A ^210^ F**	E-A	TATAAAAAA**GCA**TTAAACAGATTTGCCGATTTA
**FP2 E-A ^210^ R**	E-A	TAAATCGGCAAATCTGTTTAA**TGC**TTTTTTATA
**FP2 F-A ^214^ F**	F-A	TTAAACAGA**GCA**GCCGATTTAACTTATCAT
**FP2 F-A ^214^ R**	F-A	ATGATAAGTTAAATCGGC**TGC**TCTGTTTAA
**FP2 R-A ^185^ F**	R-A	GAAATGAAGGAA**GCA**TTTCAAGTATTC
**FP2 R-A ^185^ R**	R-A	GAATACTTGAAA**TGC**TTCCTTCATTTC
**FP3 F-A ^231^ F**	F-A	ATGAACAAA**GCA**GGAGATTTGTCCCCCGAA
**FP3 F-A ^231^ R**	F-A	TTCGGGGGACAAATCTCC**TGC**TTTGTTCAT
**FP3 E-A ^238^ F**	E-A	TCCCCCGAA**GCA**TTTAGAAGTAAATATTTAA
**FP3 E-A ^238^ R**	E-A	TTAAATATTTACTTCTAAA**TGC**TTCGGGGGA

All mutants of FP2 and FP3 were acquired by overlap extension PCR [22]. Cloning of all the mutants was accomplished as described above for the wild type genes. Purified DNA fragments were digested with *BamHI* and *HindIII* for FP2 mutants, and *BamHI* and *SalI f*or FP3 mutants. Digested products were ligated into the pQE-30 plasmid (Qiagen), and used to transform M15 (pREP4) *E.Coli* (Qiagen). The sequence of each construct was confirmed by DNA sequencing. Recombinant gene expression was induced by 1 mM IPTG and proteins were purified by nickel-nitrilotriacetic acid (Ni-NTA) chromatography under denaturing conditions as described earlier [8], [9]. Ni-NTA purified proteins were further bound to QAE-550C columns, under denaturing condition (Toyopearl), and eluted by a step wise gradient of 0–1 M NaCl in 8 M urea, 50 mM Tris, pH 8.0. The purity level of the different fractions were analyzed by 12% SDS-PAGE and visualized with Coomassie blue. The highly purified fractioned were pooled and further concentrated using a 10 kDa cut-off membrane (Vivaspin) to a final volume of 5 ml. Insoluble proteins were removed by centrifuging the sample at 21,000 g and discarding the precipitate. The denatured and purified proteins were diluted 100 fold (final concentration 20–30 µg/ml) in refolding buffer (100 mM Tris/Hcl, pH 9.0, 1 mM EDTA, 50% Glycerol, 1 mM GSH and 1.0 mM GSSG), for profalcipain-2 and its corresponding mutants. In case of profalcipain-3 and its mutants, a different refolding buffer (100 mM Tris/HCl, pH 9.0, 1 mM EDTA, 20% sucrose, 250 mM L-arginine, 1 mM GSH and 0.5 mM GSSG) was used. All samples were refolded at 4°C for 20 hr, and concentrated using a 10 kDa cut off membrane (Vivaspin).

### Amino terminal sequencing/Mass spec

Purified samples were electrophoresed in a 12% SDS- PAGE gel, stained with Coomassie blue, excised and sequenced at the CIF facility, JNU, New Delhi.

### FP2 and FP3 modeling

The 155–484 residues of FP2 (Prodomain∼155–243 and mature domain∼244–484 residues) were aligned with sequences of Procathepsin K (PDB:1BY8), Procathepsin L (PDB:1CS8), Procathepsin S (PDB:1COY), Procaricain (PDB:1PCI), and matured FP2 (PDB:1YVB). 50 homology models of FP2 were built based on these crystallographic structures. Models were evaluated with Z-DOPE score [23] and TSVMod protocol to identify the model error [24], [25]. The model with the best Z-DOPE score was used for energy minimization calculation.

The 161–491 residues of FP3 (Prodomain∼161–249, mature domain∼250–491) were aligned with sequences of Procathepsin K (PDB:1BY8), Procathepsin L (PDB:1CS8), Procathepsin S (PDB:1COY), Procaricain (PDB:1PCI), and matured FP3 (PDB:3BwKA). 50 models of FP3 were obtained using all structures as template in the MODELLER-9.10 program. Models were evaluated with Z-DOPE score and TSVMod protocol to identify the model error [24], [25]. The model with best Z-DOPE score was used for energy minimization calculation.

### Circular Dichroism analysis of wild and mutated enzyme

Experiments were performed on a ChiraScan spectropolaraimeter from Applied Photo Physics (UK) at Advanced Instrument Research facility, JNU, New Delhi. Purified proteins were concentrated (250 µg/ml) using a 10-kDa cut-off Vivaspin (Sartorius), and further exchanged with 10 mM phosphate buffer, pH 8.0. All experiments were done in a quartz cell of 1 mm path length (Hellma). CD signals were monitored between 190 and 260 nm at 25 degree celsius. CD spectra were finally measured by averaging the five best scans.

### Processing of Pro-FP2 and Pro-FP3

The processing of Pro-FP2 and Pro-FP3 was done as described earlier [8], [9]. Briefly, purified and refolded Pro-FP2; 8 µg and Pro-FP3; 6 µg, were incubated in 100 mM sodium acetate buffer, pH 5.5, 8 mM DTT at different time points (0–180 min) at room temperature. The samples were evaluated by SDS-PAGE, and western blot analysis using specific antibodies against FP2 and FP3 mature domains.

### Inhibition of processing

Purified and refolded Pro-FP2 (8 µg) was processed in the presence of different inhibitors. Leupeptin (10 µM), E-64 (4 µM) and prodomain (5 µg) were used as cysteine protease inhibitors. PMSF (phenylmethylsulfonyl fluoride; 2 mM), pepstatin (10 µM) and EDTA (1 mM), were used as serine, aspartic and mettalo proteases, respectively. Finally, proteins were evaluated by SDS-PAGE and western blot analysis using specific antibodies against the mature domain of FP2.

### Immunoblot analysis

Protein samples were resolve by SDS PAGE, and transferred to a nitrocellulose membrane (Sigma). The membranes were blocked with 1.5% BSA in PBS overnight at 4°C and incubated with anti-FP2 and anti-FP3 antibodies (1∶5000) in PBS with 0.5% BSA for 1 h at room temperature. The membranes were washed and incubated with Horse peroxidase-conjugated goat anti-rat IgG (1∶8000; Santa Cruz Biotechnolgy, California, USA) in the same solution at room temperature for 1 h. After incubation, membranes were extensively washed with PBS containing 0.2% Tween-20, and antigen–antibody complexes were developed using DAB tablets, and 0.035% of hydrogen peroxide following manufacturer's instructions (Amersco).

### Activation and Gelatin Substrate assay

Purified and refolded proteins were transferred into buffer (100 mM sodium acteate, pH 5.5, 8 mM DTT), and incubated at room temperature for 1.5 h. For native gelatin substrate PAGE, a 10% native gel (without SDS) was co-polymerized with 0.1% gelatin. The samples were incubated with loading buffer (50 mM Tris, pH 6.8, 0.1 bromophenol blue, 10% glycerol) for 10 min at room temperature and subsequently resolved in acrylamide gels using a tris-glycine buffer (25 mM Tris, 250 mM Glycine, pH 8.8). The substrate gels were incubated overnight at room temperature in 100 mM sodium acetate, pH 5.5, 8 mM DTT and visualized by Coomassie Blue staining.

### Trans activation of Pro-FP3 mutants

Different mutants were purified and refolded as previously described. Mutants of FP3 were incubated with active FP2 under optimal conditions for trans processing. For digestion, the pro-FP3 (10 µg) mutants were suspended in acetate buffer pH, 5.5, and 5 µg of active FP2 was mix with each reactions and incubated with 40 minute at room temperature. Incubations were stopped by the addition of E-64 to 10 µM. Digestion products were analyzed by SDS-PAGE and western blot analysis using specific antibodies against FP3.

## Results

### Activation of FP2

**Figure 1:**
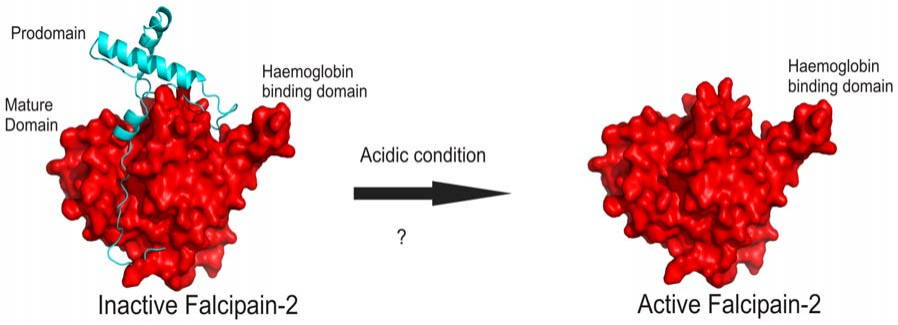
Auto-activation of Pro-FP2. The activation of Pro-FP2 was represented by complex of prodomain (cyan) and mature domain (Purple). Structural features of inactive and active FP2 were shown, where 160 N-terminal residues of the prodomain was not included in the model.

**Figure 2:**
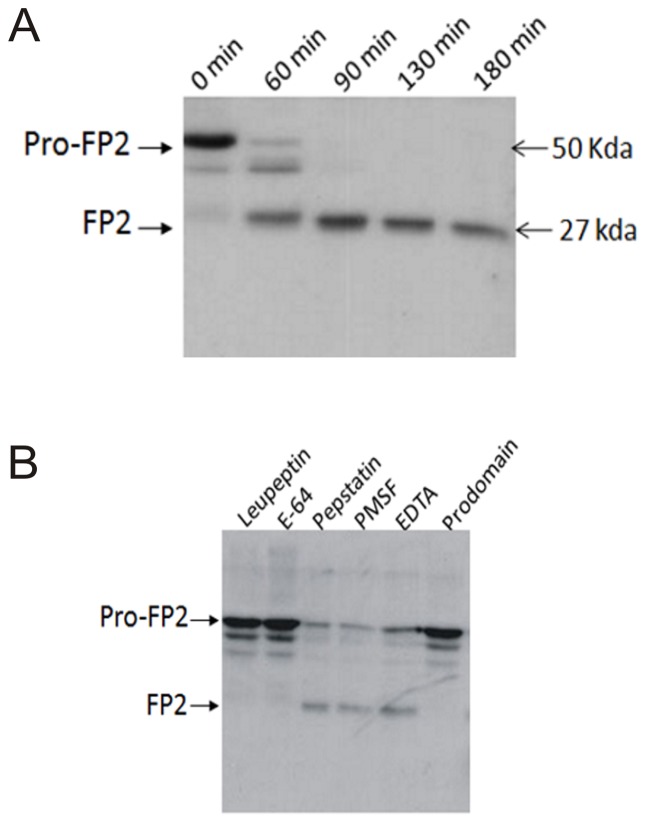
Expression, purification, refolding and processing of Pro-FP2. **A.** Purified and refolded Pro-FP2 was activated/processed in acidic condition. The processing of enzyme was followed by 3 hrs and further evaluated by SDS-PAGE and Western blot analysis. The approximate size of Pro-FP2 and FP2 were mentioned in the figure. **B.** Purified and refolded Pro-FP2 was processed in the presence of different inhibitors. Leupeptin, E-64 and, prodomain of FP2, were used as a cysteine proteases inhibitors. Whereas, PMSF (phenylmethylsulfonyl fluoride), pepstatin and EDTA were used as serine, aspartic and mettalo proteases, respectively. Finally, proteins were evaluated by SDS-PAGE and western blot analysis.

Pro-FP2 and Pro-FP3 were synthesized as inactive proenzymes, which were subsequently processed to the mature form in acidic conditions. The activation of Pro-FP2 has been demonstrated structurally (Fig. 1). Although, previous structural and biochemical studies explain the mode of inhibition, mechanism of refolding, mode of hemoglobin binding and hydrolyzing [12], [13], [16], [17], [26], but it is unknown how these proteases get activated. To investigate the optimum requirements for the processing of FP2, we expressed Pro-FP2 in *E. coli*, purified by affinity and ion exchange chromatography, refolded by rapid dilution, and evaluated for processing. The Pro-FP2 underwent processing in acidic environment, which was completed in 90 minutes (Fig. 2A). A processing intermediate was observed after 60 minutes of incubation. At 90 minutes one major band was observed resembling the mature domain of the enzyme, further confirmed by specific antibodies of FP2 (Fig. 2A).

### Effect of small and macromolecular inhibitors on the activation of Pro-FP2

Above result suggested that the processing of Pro-FP2 is self-mediated. To further substantiate that it is autocatalytic, processing was assessed in the presence of inhibitors of different classes of proteases. The small molecule cysteine protease inhibitors, leupeptin and E-64, and the macromolecule cysteine proteases inhibitors, FP2 prodomain and falstatin [27] blocked the processing (Fig. 2B, data not shown for falstatin), whereas the non-cysteine protease inhibitors pepstatin, PMSF, and EDTA did not block processing (Fig. 2B). Since the processing of the Pro-FP2 could be inhibited by cysteine proteinase inhibitors, only FP2 itself was a likely candidate to mediate its own processing. This result indicates that the free active site is essential for autocatytic activity of FP2.

### Role of conserved aspartic residue (Asp^151^) in processing of Pro-FP2

**Figure 3:**
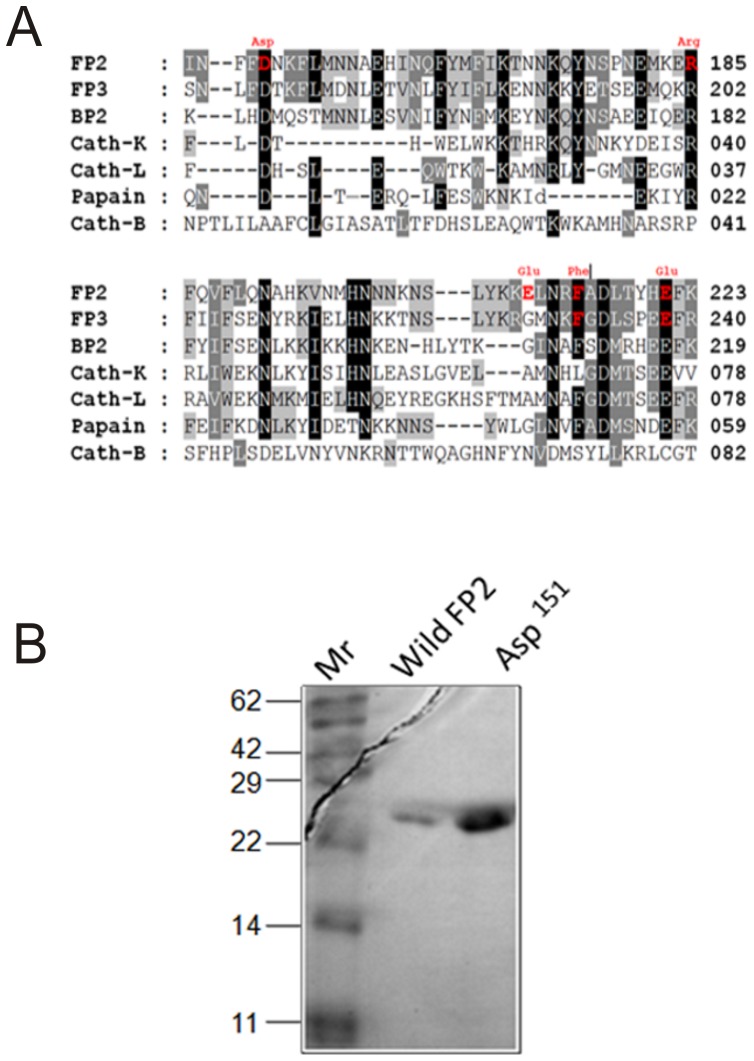
Alignment of C-terminus amino acid residues of the prodomains of FP2, FP3, and related papain family cysteine proteases. **A.** The sequences of FP2, FP3, berghepain-2 (BP2), and human cysteine proteases, cathepsin K (Cath K), cathepsin L (Cath L), cathepsin B (Cath B), and papain were aligned using Gene doc and Clustal W. The mutated amino acids are shown in red, and highly conserved amino acids are highlighted in black. **B.** The Mutant (Asp^151^) was purified and refolded and processed under optimized acidic condition as described in material methods. The protein was resolved by SDS-PAGE, and analyzed by Western blot analysis. The positions of molecular weight markers (kDa) are indicated.

The aspartic residue at position 151 in the FP2 prodomain is highly conserved within the papain family enzymes (Fig. 3A). In order to determine the role of this residue, a Pro-FP2 mutant containing Ala in place of Asp^151^ was evaluated for processing in acidic environment. We did not see any change in processing compare to the wild type (Fig. 3B). The mutant underwent processing just like the wild type FP2, indicating that Asp^151^ has no role in processing.

### Prediction of crucial residues by homology modeling

**Figure 4:**
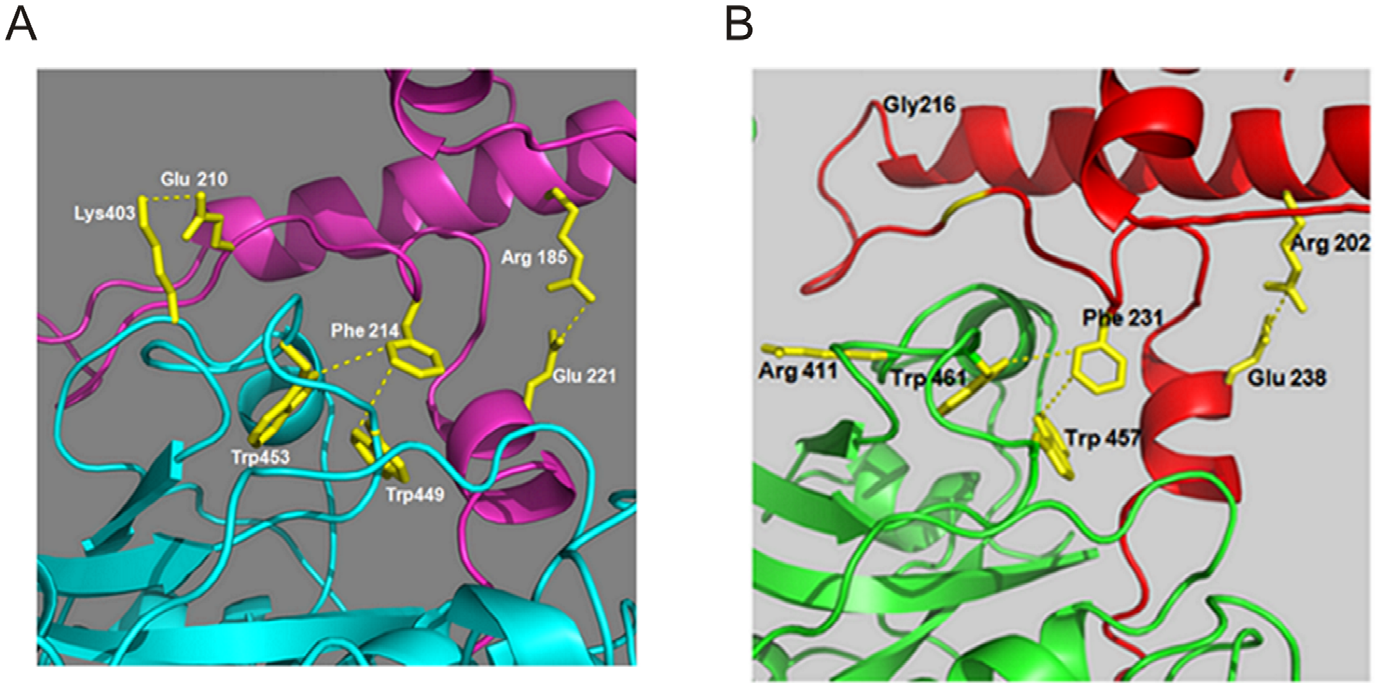
Predicted interactions between the prodomain and the mature domain of FP2 and FP3. **A.** Close up of predicted interactions between the mature enzyme and the ERFNIN and GNFD motifs of the prodomain (Arg ^185^ - Glu ^221^, and Phe ^214^-Trp^449^/Trp ^453^, Glu ^210^ - Lys ^403^). Blue dashed lines indicate presumed stabilizing interactions between residues in FP2. **B.** Blue dashed lines indicate presumed stabilizing interactions (Arg ^202^-Glu^238^ and Phe ^231^-Trp^457^/Trp461) between the residues in FP3.

We examined the homology models for possible interactions between the prodomain and the mature domain residues of FP2 and FP3 (Fig. 4). In accordance with our earlier modeling study [16] in FP2, the charged pair Arg^185^/Glu^221^ appears to form a salt bridge within the prodomain, and Glu^210^ of the GNFD motif may form another salt bridge with Lys^403^ in the mature domain. The Phe^214^ may participate in non polar and pi stacking interactions, especially with two tryptophan residues (Trp ^449^ and Trp ^453^) in the mature domain (Fig. 4A). Similarly, in the case of FP3, the charged pair Arg^202^/Glu^238^ appears to form a salt bridge within its prodomain and Phe^231^ may participate in non polar interactions with two tryptophan residues (Trp ^457^ and Trp ^461^) present in the mature domain (Fig. 4B).

### Circular dichroism analysis of wild type and mutants enzymes

**Figure 5:**
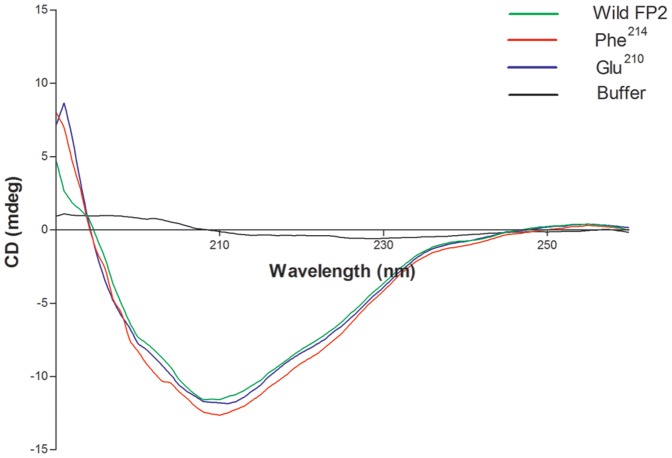
Circular dichroism analysis of wild and mutants of Pro-FP2. Wild and different mutants of Pro-FP2 (Glu ^210^ and Phe ^214^) were incubated in 10 mM phosphate buffer, pH 8.0, and absorbance between 190 and 240 nm was measured. CD spectra were finally measured after average of five best scans.

FP2 and the FP2 mutants were expressed in *E. coli*, purified by affinity and ion exchange chromatography. The proteins were refolded by rapid dilution, and evaluated by circular dichroism. CD spectroscopy was used to probe the secondary structure of refolded wild and mutants of FP2. CD spectra clearly demonstrated that the wild type FP2 and the mutants (Glu^210^ and Phe^214^) have similar secondary structures. The mutations did not alter the secondary structures, suggesting that all the mutants were properly refolded (Fig. 5).

### Identification/confirmation of crucial residues for processing of FP2 and FP3 by mutagenesis study: The role of salt bridges

**Figure 6:**
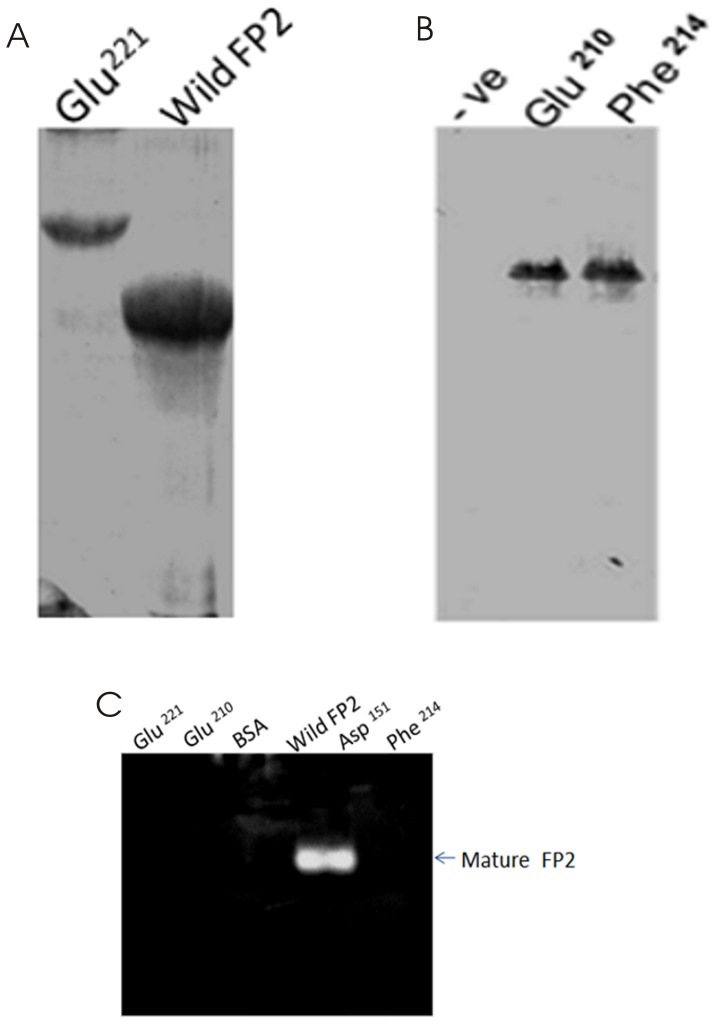
Role of salt bridge and hydrophobic interactions in FP2. **A.** The mutated enzyme (Glu ^221^) was expressed in *E.Coli*, purified and refolded, and finally processed and compare with wild FP2, analyzed by SDS-PAGE and Western blot. **B.** Two other mutants (Glu ^210^ and Phe ^214^) were also expressed in *E.coli*, purified and refolded. The processing of those mutants was further analyzed by SDS-PAGE and Western blot analysis. The uninduced *E.Coli* lysates was used as a negative control. **C.** The wild FP2 and mutated enzymes (Glu ^221^, Glu ^210^, Asp ^151^, Phe ^214^) were processed and subjected to gelatin substrate native PAGE, and assessed their functional activity.

Homology modeling based on the structure of FP2 mature domain and the prodomain structures of cathepsin K and cathepsin L have identified two salt bridges (Arg ^185^ - Glu ^221^, Glu ^210^ - Lys ^403^), which may be playing an important role in processing or activation. To validate this hypothesis, mutants were constructed in which salt bridge forming residues were replaced. These mutants were subsequently assessed for processing under acidic pH. Mutation of Glu ^221^ to Ala abolished processing into the active form (Fig. 6A). Thus, the salt bridge between Arg ^185^ and Glu ^221^ appears to play an important role during processing (Fig. 6A). To further confirm this data, another mutant was generated to eliminate the same salt bridge (Arg ^185^ - Glu ^221^) and evaluated for processing. In agreement with the previous mutant, the Arg^185^– Ala mutant was also no longer processed into the active form (data not shown), confirming that the Arg ^185^ - Glu ^221^ salt bridge is essential for Pro-FP2 processing. To evaluate the role of other salt bridge (Glu ^210^ and Lys ^403^), Glu ^210^ residue was mutated into alanine and the mutant was analyzed for processing under acidic conditions by SDS-PAGE and western blot analysis. The Glu ^210^- Ala mutant failed to undergo processing (Fig. 6B), indicating that the salt bridge between Glu ^210^ of the prodomain and Lys ^403^ of the mature domain has crucial role in processing of Pro-FP2.

**Figure 7:**
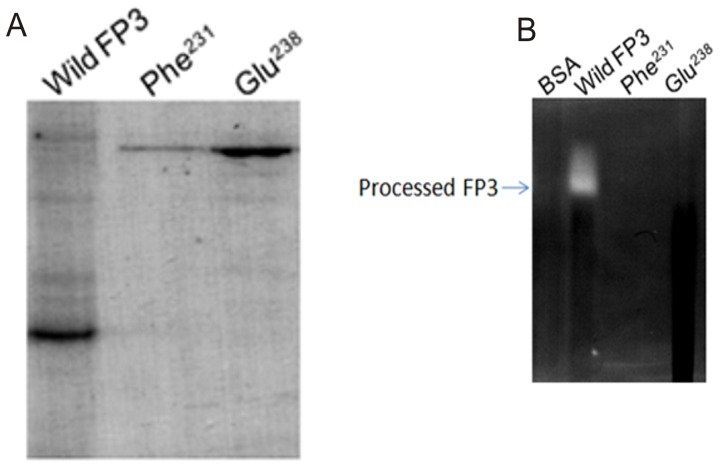
Requirements for ionic and hydrophobic interactions for processing of Pro-FP3. **A.** The wild FP3 and mutated enzyme, Glu ^238^ and Phe ^231^ were expressed in *E.Coli*, purified and refolded, and finally processed and compare with wild FP3 . The enzymes were further analyzed by SDS-PAGE and Western blot. **B.** The wild FP3 and mutated enzymes (Phe ^231^, Glu ^238^) were processed and subjected to gelatin substrate native PAGE, and assess their functional activity.

The prodomain of FP3 also contains the same conserved residues as FP2; Arg ^202^ of the prodomain appears to form a salt interaction with Glu ^238^ of the prodomain (Fig. 4B). Therefore, we also investigated the role of this salt bridge in the processing of Pro-FP3. The mutant (Glu ^238^- Ala) was expressed in *E. coli*, purified, refolded, and compared for processing with the wild type FP3 (Fig. 7A). As expected, wild type Pro-FP3 underwent processing but the mutant did not, indicating that the salt bridge (Arg ^202^-Glu ^238^) within the prodomain is crucial for processing (Fig. 7A).

### Role of hydrophobic interactions

The Pro-FP2 and Pro-FP3 models suggested that conserved residues provide stability by non-polar interactions. In case of Pro-FP2, Phe ^214^ of the prodomain appears to form hydrophobic interaction with the Trp ^449^ and Trp ^453^ of the mature domain (Fig. 4A). Similarly, the Phe^231^ residue of the prodomain of FP3 also appears to interact with the Trp^457^ and Trp^461^ residues of the mature domain (Fig. 4B). To determine if hydrophobic interactions are required for processing, Phe ^214^ and Phe ^231^ mutants of FP2 and FP3 were generated respectively, and further assessed for processing. Both these mutants could not be processed (Fig. 6B, Fig. 7A), indicating that hydrophobic interactions mediated by these residues are crucial for processing.

### Functional analysis of wild type enzymes and different mutants of FP2 and FP3

To analyze the proteases activity of wild type and mutants of FP2 and FP3, gelatin zymography assay was performed. The Asp^151^, even though it is a highly conserved charged residue, did not affect processing, and showed hydrolysis of gelatin at the size of the wild type FP2, thus again confirmed that Asp^151^ is not required for processing (Fig. 6C). On the other hand, we also analyzed the functional assay of all the mutants of FP2 (Glu ^221^, Glu ^210^, Phe ^214^) and FP3 (Phe ^214^, Glu ^231^), as expected mutants did not show any processing, which is further confirmed by gelatin zymography functional assay (Fig. 6C and Fig. 7B).

### Trans processing of Pro-FP3 mutants

**Figure 8:**
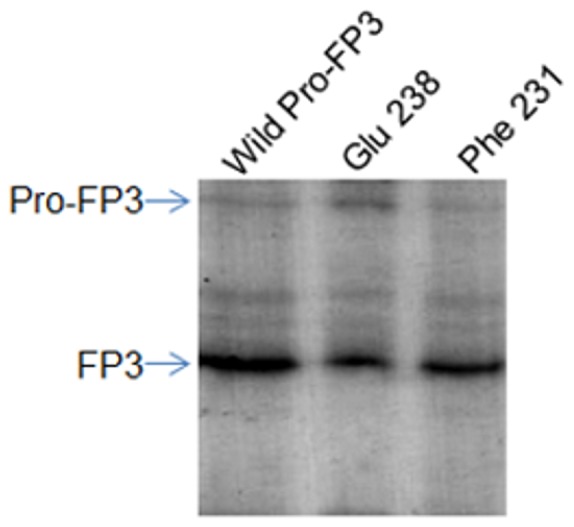
Trans processing of the Pro-FP3 mutant. Mutants of FP3 were incubated with active FP2 under optimal conditions for trans processing. For digestion, the pro-FP3 mutants (10 µg) were suspended in acetate buffer and 5 µg of active FP2 was mix with each reaction and incubated at room temperature. The wild Pro-FP3, which was auto processed used as control. Digestion products were analysed by SDS-PAGE and western blot analysis using specific antibodies against FP3.

To see the effect of active falcipain on its non- active precursors, different mutants of pro-FP3 were incubated with active FP2, and processing was followed under optimal conditions. Western blot analysis clearly showed that mutants could be processed in trans (Fig. 8). This result further supported the CD data that mutants also have similar folds as compare to wild enzyme, and explained that the hydrophobic and salt interactions are specifically responsible for auto processing.

## Discussion

Falcipain-2 and falcipain-3 are two major papain-like cysteine proteases of *P. falciparum* with roles in hemoglobin hydrolysis, merozoite egress, and processing of gametocyte surface proteins. These two enzymes have unique motifs for mediating activity, transport to the food vacuole, inhibition of the cognate mature enzymes, and binding to hemoglobin. However, it is not known how these enzymes get processed, a major step in which the inhibitory prodomain is cleaved and releasing the mature enzyme for hemoglobin hydrolysis. We predicted several interactions between the prodomain and the mature domains, and generated mutants lacking the amino acid residues participating in interactions, and assessed processing of mutants to determine the mechanism of activation of falcipain-2 and falcipain-3. Our results provide unambiguous evidence for essential roles of salt bridge and hydrophobic interactions between the prodomain and the mature domain residues in auto processing.

Our initial study based on highly conserved residues within the papain family cysteine proteases; we designed to mutate, Asp^151^, but did not show any effect on activation. This negative charge residue is highly conserved and present in all papain family enzymes, except cathepsin B. Our first mutant (Asp^151^) result suggests that although it is a conserved residue it may not be crucial for processing because it is not directly taking part in any interactions between the domains.

Further, designing the mutants based on structures of falcipain-2 and falcipain-3, and cathepsin K, L, we identified different interactions which might be playing a crucial role in the activation of enzymes. Since the wild type and mutated enzymes were expressed as inclusion bodies, purified proteins were refolded and their folding patterns were analyzed. CD spectra showed that wild type falcipain-2 and mutants have similar secondary structures, suggesting that all the mutants are properly folded. In fact our previous data also showed that the refolding domain of enzyme is present at the N-terminal of the mature domain [12], which is separate and independent from mutated residues and that can be a reason why mutated residues do not alter the secondary structure of mutants, and refolded like wild enzyme. Since the refolding domain and mutated interactions are functionally conserved in both the enzymes, FP3 mutants are likely to refold in a similar way as FP2 mutants. Further, to validate the refolding of FP3 mutants, trans activation experiment showed that FP3 mutants also have similar folds as wild FP3, and can be activated in trans.

There are salt bridges, Arg^185^-Glu ^221^ and Arg ^202^-Glu ^238^ in falcipain-2 and falcipain-3 respectively, which are present within the prodomains. Mutagenesis studies were done to abolish these interactions and find out whether these interactions are crucial for activation of falcipain-2 and falcipain-3. Abolishing these salt bridges probably affect the release of the prodomain required for activation. We observed two kinds of interactions in between the prodomain and the mature domain of falcipain-2. First, a hydrophobic core is formed between the prodomain and the mature domain by interacting Phe ^214^,Trp ^449^ and Trp ^451^. Second, a salt bridge is form between the prodomain (Glu ^210^) and the mature domain (Lys ^403^). Abolishing the hydrophobic and ionic interactions, mutagenesis study clearly shows that pro-enzymes can no longer process into active enzyme. These results suggest that these interactions are crucial for the stability of the pro-falcipain-2 complex. Together, there are three possible interactions (two ionic and one hydrophobic), the first ionic interaction within the prodomain seems essential for stabilizing the prodomain for processing event. The other two (ionic and hydrophobic) interactions between the prodomain and the mature domain may also play an important role by stabilizing the whole complex, which may be prerequisite for the successful activation of enzymes. Our data suggest that a stable complex of the whole enzyme is required for successful activation. Once the interactions between the prodomain-mature domains are disturbed, the released of the prodomain may not be possible for successful processing. A similar study was conducted for falcipain-3. Here, two important interactions, a salt bridge (Arg ^202^-Glu ^238^) and a hydrophobic interaction (Phe ^231^,Trp ^457^, Trp ^461^) are essential for the auto activation.

**Figure 9:**
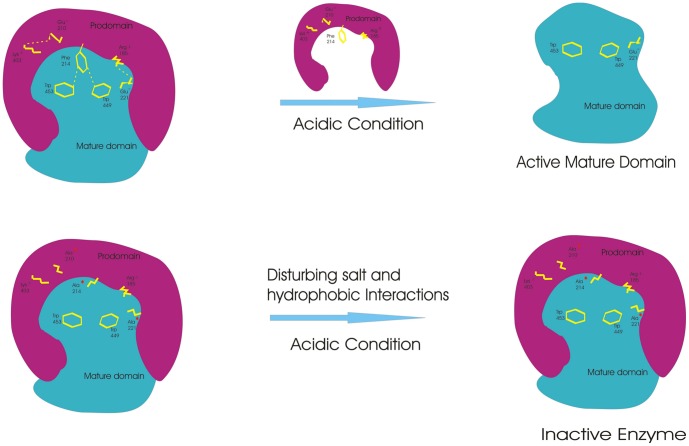
Model showing mechanism of activation of falcipain. The prodomain (purple) and the mature domain (cyan) are stabilized by salt and hydrophobic interactions (shown by yellow dashes). The wild enzyme (upper panel) processed normally in acidic condition where the prodomain is released and the mature domain is catalytically active. On the other hand, mutants (mutations indicated by red labeled) with disrupted salt and hydrophobic interactions failed to process into active enzyme.

The processing of Pro-FP3 mutants clearly showed that mutants could be processed in trans. This result further supported the CD data that mutants also have similar folds as compare to wild enzyme, and explained that the hydrophobic and salt interactions are specifically required for auto processing. Based on our mutagenesis study, we proposed a model (Fig. 9) that explains the prodomain and the mature domain interactions are necessary for successful auto activation of FP2 and FP3.

Residues that appear to be involved in these interactions are quite well conserved within falcipain-2 and falcipain-3, but interestingly they are also involved in domain-domain interactions [16]. The sequence alignment data indicate that residues, which are responsible for the prodomain-mature domain interactions are conserved in all papain family proteases except cathepsin B (Fig. 3A). Like other cysteine proteases, cathepsin B is also processing by autocatalytic activity [18], [19], [20]. Although, in vitro experiments showed that procathepsin B could also be activated by the action of different proteases [28]. However, the basic mechanism is unknown. It has been shown that the inhibition of cathepsin B by its prodomain is different to other class of the papain subfamily [16], [29], [30]. It lacks the ERFNIN motifs, which results in the lack of most of the α 2 helix, and it contains a large occluding loop insertion, conferring with exopeptidase activity, which is a peculiar character of this papain family enzyme. Human cathepsin D, an aspartic protease processes differently compare to other cathepsins and falcipains. Unlike, falcipains and cathepsins K, L and B, the processing of cathepsin D is an independent of its catalytic function and auto activation [31]. It is likely that cathepsin D has a different way of processing as compared to the papain family cysteine proteases.

As previously described in other proteases, processing occurs in acidic environments, and just like other papain family proteases, falcipains can be processed by autocatalytic activity. Our result suggests that autocatalytic activity of falcipains can be blocked by both small and macromolecular cysteine protease inhibitors. In this study, we have shown that the prodomain of falcipain-2, and falstatin, an endogenous cysteine protease inhibitor present in *P.falciparum*, are two major macromolecular inhibitors of auto processing. It seems logical that the prodomain and falstatin function to control the activities of parasite proteases. These endogenous inhibitors serving their role to prevent inappropriate protease activity may be crucial for parasite survival.

It is interesting to raise a question, how a pH drop may trigger processing of the proteases? Based on the 3D structure of pro-cathepsin-B, it was suggested that an early conformational change in the propeptide may be induced by acidic environment [32]. This was further supported by Rozman et al, where pH-induced conformational changes were observed in the propeptide of cathepsin L [33]. However, no conformational changes were observed during the autoactivation of cathepsin L and cathepsin B in other studies [28], [34]. As discussed above the processing of cathepsin B and cathepsin L occur at acidic pH without significant conformational changes suggesting that propeptide unfolding occur only after the cleavage. According to previous study, the activation process is triggered by pH drop that presumably weakening the interactions between the propeptide and the catalytic domain [35]. As a result, the propeptide may have a looser conformation and as a consequence of that the propeptide bound less tightly into the active site without loss of the secondary structure [28], [34]. Such state of enzyme appears to exhibit catalytic activity sufficient to initiate the chain reaction. However, the detail mechanism of initiation of processing still remains an intriguing part. It would be interesting to study the 3D structures of different states of proenzymes at acidic environment.

Our results of all the mutants' were straight forward, revealing clear structural and functional relationship of FP2 and FP3 auto processing. The mutant based on the highly conserved residue (Asp^151^) within the papain family did not have any effect on processing. This might be due to no direct involvement of this residue in either interacting within the prodomain or prodomain-mature domain interactions. Our modeling study based on crystal structures of falcipain-2 and falcipain-3, further revealed that Asp ^151^ residue is not involved in any of these interactions, which we have mentioned above. Indeed, on the other hand, residues directly interacting as salt bridges and hydrophobic interactions play crucial roles in activation of enzymes. Taken together, our results identify the mechanism of auto activation of major cysteine proteases of *P.falciparum*.

## Conclusion

The prodomain-mature domain interactions are necessary for the auto-activation of FP2 and FP3.

## References

[1] WHO World Malaria Report (2008).

[2] Fidock DA (2010) Drug discovery; Priming the antimalarial pipeline. Nature 465: 297–298. 20485420 10.1038/465297a

[3] Wongsrichanalai C, Meshnick SR (2008) Declining artesunate-mefloquine efficacy against falciparum malaria on the Cambodia-Thailand border. Emerg Infect Dis 14: 716–719. 18439351 10.3201/eid1405.071601 PMC2600243

[4] Dondorp AM, Nosten F, Yi P, Das D, Phyo AP, et al (2009) Artemisinin resistance in *Plasmodium falciparum* malaria. N Engl J Med 361: 455–67. 19641202 10.1056/NEJMoa0808859 PMC3495232

[5] Rosenthal PJ, Sijwali PS, Singh A, Shenai BR (2002) Cysteine proteases of malaria parasites: targets for chemotherapy. Curr Pharm Des 8: 1659–72. 12132997 10.2174/1381612023394197

[6] Rosenthal PJ (2004) Cysteine proteases of malaria parasites. Intl J Parasitology 34: 1489–1499. 10.1016/j.ijpara.2004.10.003 15582526

[7] Shenai BR, Sijwali PS, Singh A, Rosenthal PJ (2000) Characterization of native and recombinant falcipain-2, a principal trophozoite cysteine protease and essential hemoglobinase of *Plasmodium falciparum* . J Biol Chem 37: 29000–29010. 10.1074/jbc.M004459200 10887194

[8] Sijwali PS, Shenai BR, Gut J, Singh A, Rosenthal PJ (2001) Expression and characterization of the *Plasmodium falciparum* haemoglobinase falcipain-3. Biochem J 360: 481–489. 11716777 10.1042/0264-6021:3600481 PMC1222249

[9] Sijwali PS, Shenai BR, Rosenthal PJ (2002) Folding of the *Plasmodium falciparum* cysteine protease falcipain-2 is mediated by a chaperone-like peptide and not the prodomain. J Biol Chem 277: 14910–14915. 11827964 10.1074/jbc.M109680200

[10] Sijwali PS, Rosenthal PJ (2004) Gene disruption confirms a critical role for the cysteine protease falcipain-2 in haemoglobin hydrolysis by *Plasmodium falciparum* . Proc Natl Acad Sci USA 13: 4384–4389. 10.1073/pnas.0307720101 PMC384756 15070727

[11] Sijwali PS, Koo J, Singh N, Rosenthal PJ (2006) Gene disruptions demonstrate independent roles for the four falcipain cysteine proteases of *Plasmodium falciparum* . Mol Biochem Parasitol 150: 96–106. 16890302 10.1016/j.molbiopara.2006.06.013

[12] Pandey KC, Sijwali PS, Singh A, Na BK, Rosenthal PJ (2004) Independent intramolecular mediators of folding, activity, and inhibition for the *Plasmodium falciparum* cysteine protease falcipain-2. J Biol Chem 5: 3484–3491. 10.1074/jbc.M310536200 14625277

[13] Pandey KC, Wang SX, Sijwali PS, Lau AL, McKerrow JH, et al (2005) The *Plasmodium falciparum* cysteine protease falcipain-2 captures its substrate, haemoglobin, via a unique motif. Proc Natl Acad Sci USA 102: 9138–9143. 15964982 10.1073/pnas.0502368102 PMC1166610

[14] Pandey KC, Dixit R (2011) Structure-Function of Falcipains; Malarial Cysteine Proteases. Invited review in Journal of Tropical Medicine 10.1155/2012/345195. 10.1155/2012/345195 PMC3317066 22529862

[15] Subramanian S, Sijwali PS, Rosenthal PJ (2007) Falcipain cysteine proteases require bipartite motifs for trafficking to the *Plasmodium falciparum* food vacuole. J Biol Chem 282: 24961–24969. 17565983 10.1074/jbc.M703316200

[16] Pandey KC, Barkan DT, Sali A, Rosenthal PJ (2009) Regulatory elements within the prodomain of Falcipain-2, a cysteine protease of the malaria parasite *Plasmodium falciparum* . PLoS One 4e: 5694. 10.1371/journal.pone.0005694 PMC2682653 19479029

[17] Subramanian S, Hardt M, Choe Y, Niles RK, Johansen EB, et al (2009) Haemoglobin cleavage site-specificity of the *Plasmodium falciparum* cysteine proteases falcipain-2 and falcipain-3. PLoS One 4e: 5156. 10.1371/journal.pone.0005156 PMC2663817 19357776

[18] Nishimura Y, Amano J, Sato H, Tsuji H, Kato K (1988) Biosynthesis of lysosomal Cathepsins B and H in cultured rat hepatocytes. Arch Biochem & Biophys 262: 159–170. 3128174 10.1016/0003-9861(88)90178-6

[19] Kominami E, Tsukahara T, Hara K, Katunuma N (1988) Biosynthesis and processing of lysosomal cysteine proteases in rat macrophages. FEBS Lett 231: 225–228. 3360126 10.1016/0014-5793(88)80736-1

[20] Rojman J, Stojan J, Kuhelji R, Turk V, Turk B (1999) Autocatalytic Processing of human procathepsin B is a bimolecular process. FEBS Lett 459: 358–362. 10526165 10.1016/s0014-5793(99)01302-2

[21] Dahl EL, Rosenthal PJ (2005) Biosynthesis, localization, and processing of falcipain cysteine proteases of *Plasmodium falciparum* . Mol Biochem Parasitol 139: 205–212. 15664655 10.1016/j.molbiopara.2004.11.009

[22] Ho SN, Hunt HD, Horton RM, Pullen JK, Pease LR (1989) Site-directed mutagenesis by overlap extension using the polymerase chain reaction. Gene 77: 51–59. 2744487 10.1016/0378-1119(89)90358-2

[23] Shen MY, Sali A (2006) Statistical potential for assessment and prediction of protein structures. Protein Sci 15: 2507–2524. 17075131 10.1110/ps.062416606 PMC2242414

[24] Eramian D, Eswar N, Shen MY, Sali A (2008) How well can the accuracy of comparative protein structure models be predicted? Protein Science 17: 1881–93. 18832340 10.1110/ps.036061.108 PMC2578807

[25] Fiser A, Do RK, Sali A (2000) Modeling of loops in protein structures. Protein Science 9: 1753–1773. 11045621 10.1110/ps.9.9.1753 PMC2144714

[26] Wang SX, Pandey KC, Somoza JR, Sijwali PS, Kortemme T, et al (2006) Structural basis for unique mechanisms of folding and haemoglobin binding by a malarial protease. Proc Natl Acad Sci USA 31: 11503–11518. 10.1073/pnas.0600489103 PMC1544199 16864794

[27] Pandey KC, Singh N, Arastu Kapur S, Bogyo M, Rosenthal PJ (2006) Falstatin, a cysteine protease inhibitor of *Plasmodium falciparum*, facilitates erythrocyte invasion. PLoS Pathogen 11: e117. 10.1371/journal.ppat.0020117 PMC1630708 17083274

[28] Cygler M, Sivaraman J, Grochulski P, Coulombe R, Storer AC, et al (1996) Structure of rat procathepsin B: model for inhibition of cysteine protease activity by the proregion. Structure 4: 405–416. 8740363 10.1016/s0969-2126(96)00046-9

[29] Turk D, Podobnik M, Kuhelj R, Dolinar M, Turk V (1996) Crystal structures of human procathepsin B at 3.2 and 3.3 angstroms resolution reveal an interaction motif between a papain-like cysteine protease and its propeptide. FEBS Lett 384: 211–214. 8617355 10.1016/0014-5793(96)00309-2

[30] Groves MR, Coulombe R, Jenkins J, Cygler M (1998) Structural basis for specificity of papain –like cysteine protease proregions toward their congate enzymes. Proteins 32: 504–514. 9726419

[31] Laurent-Matha V, Derocq D, Prebois C, Katunuma N, Liaudet-Coopman E (2006) Processing of human cathepsin D is independent of its catalytic function and auto-activation: involvement of cathepsins L and B. J Biochem 139: 363–371. 16567401 10.1093/jb/mvj037 PMC2376303

[32] Podobnik M, Kuhelj R, Turk V, Turk D (1997) Crystal structure of the wild-type human procathepsin B at 2.5 A resolution reveals the native active site of a papain-like cysteine protease zymogen. J Mol Biol 271: 774–788. 9299326 10.1006/jmbi.1997.1218

[33] Jerala R, Zerovnik E, Kidric J, Turk V (1998) pH-induced conformational transitions of the propeptide of human cathepsin L. A role for a molten globule state in zymogen activation. J Biol Chem 273: 11498–11504. 9565563 10.1074/jbc.273.19.11498

[34] Ménard R, Carmona E, Takebe S, Dufour E, Plouffe C, et al (1998) Autocatalytic processing of recombinant human procathepsin L. Contribution of both intermolecular and unimolecular events in the processing of procathepsin L in vitro. J Biol Chem 273: 4478–4484. 9468501 10.1074/jbc.273.8.4478

[35] Fox T, de Miguel E, Mort JS, Storer AC (1992) Potent slow-binding inhibition of cathepsin B by its propeptide. Biochemistry 31: 12571–12576. 1472493 10.1021/bi00165a005

